# Clinical and genetic analysis of Vietnamese patients diagnosed with early‐onset Parkinson's disease

**DOI:** 10.1002/brb3.2950

**Published:** 2023-03-06

**Authors:** Minh Duc Do, Tai Ngoc Tran, An Bac Luong, Linh Hoang Gia Le, Tuan Van Le, Khuong Thai Le, Niem Thanh Van Vo, Thuc‐Nhi Nguyen Le, Hoang Anh Vu, Thao Phuong Mai

**Affiliations:** ^1^ Center for Molecular Biomedicine University of Medicine and Pharmacy at Ho Chi Minh City Ho Chi Minh City Vietnam; ^2^ Movement Disorder Unit, Department of Neurology University Medical Center University of Medicine and Pharmacy at Ho Chi Minh City Ho Chi Minh City Vietnam; ^3^ Department of Neurology Faculty of Medicine University of Medicine and Pharmacy at Ho Chi Minh City Ho Chi Minh City Vietnam; ^4^ Department of Physiology‐Pathophysiology‐Immunology, Faculty of Medicine University of Medicine and Pharmacy at Ho Chi Minh City Ho Chi Minh City Vietnam

**Keywords:** genetic, multiplex ligation‐dependent probe amplification, next‐generation sequencing, Parkinson's disease, Vietnam

## Abstract

**Background:**

Genetic factors play a crucial role in the pathogenesis of Parkinson's disease (PD). However, no comprehensive study has described genetic alterations in Vietnamese patients diagnosed with PD. This study aimed to identify genetic causes and their association with clinical phenotypes in a Vietnamese PD cohort.

**Methods:**

A total of 83 patients with early‐onset PD (disease onset before the age of 50) were recruited for genetic analysis using a combination of multiplex ligation‐dependent probe amplification and next‐generation sequencing for a panel of 20 PD‐associated genes.

**Results:**

It was found that 37 out of 83 patients carried genetic alterations, with 24 pathogenic/likely pathogenic/risk variants and 25 variants of uncertain significance. The pathogenic/likely pathogenic/risk variants were mostly detected in *LRRK2*, *PRKN*, and *GBA*, while the variants of uncertain significance were identified in 12 different genes that were studied. The most common genetic alteration was *LRRK2* c.4883G>C (p.Arg1628Pro), and patients with PD carrying this variant were found to have a distinct phenotype. Participants carrying pathogenic/likely pathogenic/risk variants had a significantly higher rate of a family history of PD.

**Conclusion:**

These results provide a further understanding of genetic alterations associated with PD in a South‐East Asian population.

## Graphical abstract

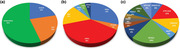

## 1 INTRODUCTION

Parkinson's disease (PD) is one of the most common neurodegenerative diseases and is characterized clinically by bradykinesia, resting tremor, rigidity, and posture instability (De Lau & Breteler, 2006; Kalia & Lang, 2015). The disease is estimated to affect 0.3% of the general population, and its prevalence increases with age (Pringsheim et al., 2014). The pathophysiology of PD is determined mainly by the progressive loss of dopaminergic neurons in the substantia nigra; this is a complex process influenced by both environmental and genetic factors. To date, more than 20 genes have been found to be associated with PD, and most of the genetic alterations impact early‐onset PD (EOPD), which is generally defined as disease onset before the age of 50 (Alcalay et al., 2010; Lin et al., 2019; Selvaraj & Piramanayagam, 2019). Many studies have been conducted to identify the causative genetic factors behind EOPD, as this information provides biological insights into disease pathophysiology and even helps to identify potential treatment targets (Alcalay et al., 2010; Cristina et al., 2020; Lin et al., 2019). In line with other genetic diseases, the genetic causes of PD may differ between ethnicities; therefore, expanding the molecular understanding of PD in diverse populations is crucial. The Vietnamese population has been shown to have a distinct genetic profile in terms of variant distribution and disease association (M. D. Do et al., 2021; Do et al., 2020; Tran et al., 2021; Truong et al., 2022); however, very little information regarding the genetic causes of PD has been published. In two recent studies, only three causative genes for EOPD were examined, mainly due to the limitations of the sequencing technique (Giang et al., 2017; Ton et al., 2020). Furthermore, there have been no investigations into the genetic rearrangements in PD, although they have been reported to be a potential causative factor in EOPD. Therefore, this study was designed to identify the genetic causes of EOPD by using a combination of multiplex ligation‐dependent probe amplification (MLPA) and next‐generation sequencing (NGS) for a panel of 20 PD‐associated genes: *SNCA, PRKN, GBA1, PINK1, DJ‐1, LRRK2, ATP13A2, VPS35, UCHL1, PLA2G6, FBXO7, DNAJC6, SYNJ1, HTRA2, EIF4G1, DNAJC13, CHCHD2, VPS13C, GCH1*, and *MAPT*.

## 2 MATERIALS AND METHODS

### 2.1 Subjects

A total of 83 unrelated patients diagnosed with PD before the age of 50 were recruited for this study. The study protocol was approved by the Ethical Committee of the University of Medicine and Pharmacy at Ho Chi Minh City (approval number 352/DHYD‐HDDD). The diagnosis of PD was based on the International Parkinson and Movement Disorder Society Clinical Diagnostic Criteria for Parkinson's disease (Postuma et al., 2015), with examinations by two independent Movement disorder neurologists from Movement disorder unit, Neurology Department, University Medical Center, Ho Chi Minh City. MDS‐Unified Parkinson's Disease Rating Scale (MDS‐UPDRS) and Hoehn‐Yahr scale were used to measure the progression, severity, and stage of the disease. Cognitive screening was further evaluated by Mini‐Mental State Examination (MMSE), and Montreal Cognitive Assessment (MoCA). Patients provided written informed consent upon participating in the study. Demographic and clinical information on all the participants was documented. Two milliliters of peripheral blood was collected from each patient by EDTA Vacutainer (Becton Dickinson, NJ, USA), and genomic DNA was subsequently extracted from blood samples by QIAamp DNA Blood Mini Kit (QIAGEN, Hilden, Germany) according to the manufacturer's instruction.

### 2.2 Genetic analysis

#### 2.2.1 Multiplex ligation‐dependent probe amplification

A SALSA MLPA Probemix P051‐D2 and P052‐D2 Parkinson kit (MRC‐Holland, Amsterdam, the Netherlands) were used to determine genetic rearrangements. These two kits contain probes for detecting deletions or duplication in *SNCA, PARK2, UCHL1, PINK1, DJ‐1, ATP13A2, LRRK2, GCH1* genes, and the presence of two‐point mutations, *SNCA* p.Ala30Pro and *LRRK2* p.Gly2019Ser. Fifty nanograms of gDNA was denatured and allowed to hybridize with two sets of Probemix at 60°C for 18h. Ligase enzymes were added and incubated at 54°C for 15min. The solution was subsequently amplified by PCR and electrophoresis was performed by ABI 3500 (Applied Biosystems, Waltham, MA, USA). Genetic rearrangement was analyzed using Coffalyser software. Based on the fluorescence intensity, dosage quotients (DQ) for each probe were calculated. Samples were taken to be duplications when DQ>1.3 and deletions when DQ<0.65.

#### 2.2.2 Next‐generation sequencing

The gDNA with a concentration equal to or greater than 3.0ng/μL was fragmented into 100–250 base pairs and purified. NEBNext® Ultra™ II DNA Library Prep Kit for Illumina® (New England Biolabs, Ipswich, MA, USA) was used to prepare the NGS library following the manufacturer's instructions. Equal amounts of libraries were pooled together and hybridized with xGen Lockdown probes for 20 genes: *SNCA, PRKN, GBA1, PINK1, DJ‐1, LRRK2, ATP13A2, VPS35, UCHL1, PLA2G6, FBXO7, DNAJC6, SYNJ1, HTRA2, EIF4G1, DNAJC13, CHCHD2, VPS13C, GCH1*, and *MAPT* (IDT Corporation, NJ, USA). The concentration was diluted to 2 nM measured by a Qubit 4 fluorometer (Thermo Fisher Scientific, Waltham, MA, USA). The sequencing process was performed by MiniSeq High output kits v2 (150 cycles) (Illumina, San Diego, CA, USA) on an Illumina MiniSeq system (Illumina) with a calculated minimum coverage of 40X. Basespace sequencing hub (Illumina) was used to identify genetic variants, which were designated following the recommendations of the American College of Medical Genetics (ACMG) and ClinVar (Landrum et al., 2018; Richards et al., 2015).

#### 2.2.3 Direct sequencing

Pathogenic mutations identified by NGS were subsequently confirmed by direct sequencing. Appropriate primers were designed, and the protocol used for direct sequencing was as described previously (M. D. Do et al., 2020; Kiet et al., 2019; Mai et al., 2019).

### 2.3 Statistical analysis

The clinical characteristics of the studied population were statistically analyzed using Student's *t*‐test for comparing two mean values, ANOVA one‐way test for comparing differences between the means of the groups, and Chi‐square test and Fisher's exact test for testing independence. A *p*‐value<.05 was considered statistically significant.

## 3 RESULTS

### 3.1 Clinical characteristics of patients with EOPD

**FIGURE 1:**
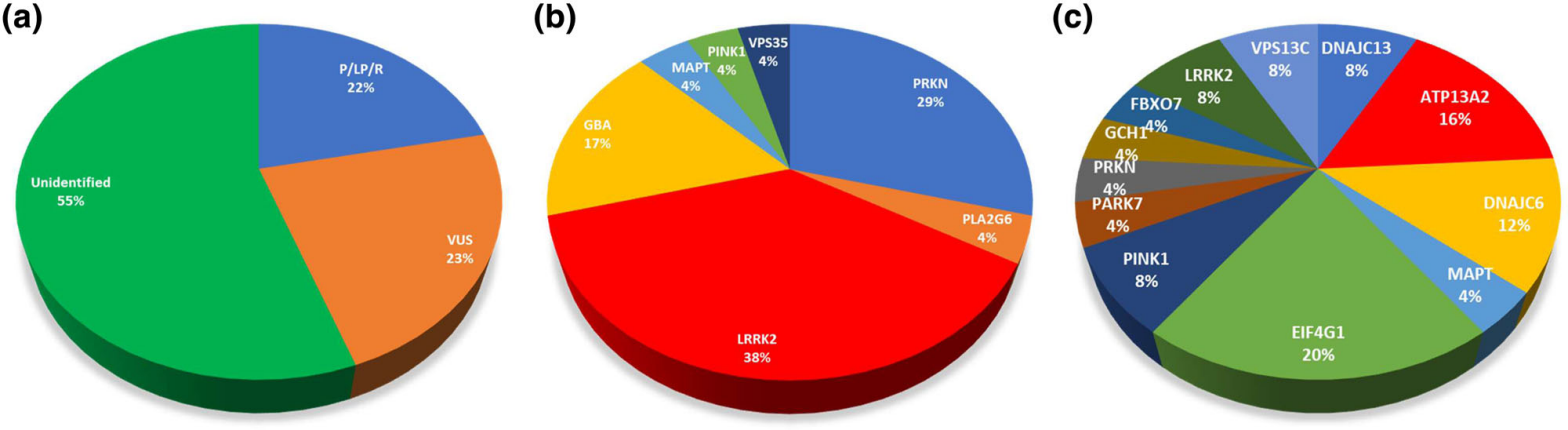
Distribution of genetic alterations in studied population. (a) Percentage of patients harboring genetic alterations in 20 genes (N=83). (b) Distribution of pathogenic alleles detected by genes (N=24). (c) Distribution of VUS alleles detected by genes (N=25). P/LP/R: pathogenic/likely pathogenic/risk variant; VUS, variant of uncertain significance.

The mean age of patients recruited in this cohort was 48.9 with a mean age of onset of PD of 43.1; 44.6% of patients were female. Most of the patients were non‐smokers, and only 12 out of 83 patients had a family history of PD. The clinical characteristics of patients with PD are summarized in Table 1, including Hoehn‐Yahr stage and MDS‐UPDRS score. Patients carrying both VUS and pathogenic/likely pathogenic/risk variants were stratified into pathogenic/likely pathogenic/risk group. The percentages of patients in the pathogenic/likely pathogenic/risk, VUS, and unidentified groups are illustrated in Figure 1a. The statistical analysis found no significant difference between the three groups of participants in all variables except for a family history of PD; patients carrying pathogenic/likely pathogenic/risk variants had a significantly higher rate of family history of PD.

**TABLE 1:** Clinical characteristics of patients with early‐onset Parkinson's disease (EOPD)

Characteristics	Total N=83	P/LP/R N=18	VUS N=19	Unidentified N=46	*p*‐Value^a^
Age, (mean±SD)	48.93±7.70	46.50±7.43	50.84±6.30	49.11±8.21	.22
Age of onset, (mean±SD)	43.14±5.97	41.55±6.10	44.00±5.32	43.41±6.18	.42
Gender, N (%)					
Male	46 (55.42)	12 (66.67)	12 (63.16)	22 (47.82)	.29
Female	37 (44.58)	6 (33.33)	7 (36.84)	24 (52.17)	
Smoking, N (%)					
Often	3 (3.61)	2 (11.11)	0	1 (2.17)	.18
Used to	22 (26.51)	4 (22.22)	8 (42.11)	10 (21.78)	
No	58 (69.88)	12 (66.67)	11 (57.89)	35 (76.08)	
Exposure to pesticides, N (%)					
Yes	28 (33.73)	5 (27.78)	6 (31.58)	17 (36.95)	.76
No	55 (66.27)	13 (72.22)	13 (68.42)	29 (63.05)	
Family history of PD, N (%)					
Yes	12 (14.45)	5 (27.78)	0	7 (15.22)	.03^b^
No	71 (85.54)	13 (72.22)	19 (100.00)	39 (84.78)	
Hoehn‐Yahr stage, N (%)					
1	15 (18.07)	5 (25.00)	3 (15.79)	7 (15.22)	.86
2	46 (55.42)	8 (50.00)	11 (57.89)	27 (58.70)	
3	21 (25.31)	5 (22.00)	5 (26.32)	11 (23.91)	
4	1 (1.20)	0	0	1 (2.17)	
MDS‐UPDRS part I (mean±SD)	7.55±4.68	6.55±3.84	7.47±5.24	7.98±4.77	.55
MDS‐UPDRS part II (mean±SD)	11.05±6.10	11.06±6.54	11.95±6.70	10.67±5.75	.75
MDS‐UPDRS part III (mean±SD)	29.77±14.93	29.39±17.59	26.21±16.36	31.39±13.20	.44
MDS‐UPDRS part IV (mean±SD)	3.11±3.48	3.22±3.94	3.68 ± 3.59	2.82±3.29	.66

Abbreviations: EOPD, early‐onset Parkinson's disease; MDS‐UPDRS, MDS‐Unified Parkinson's Disease Rating Scale; P/LP/R, pathogenic/likely pathogenic/risk variant; PD, Parkinson's disease; VUS, variant of uncertain significance.

^a^ *p*‐Value for statistical analysis between three groups (P/LP/R, VUS, and Unidentified).

^b^ Statistically significant.

### 3.2 Molecular characteristics of patients with EOPD

The molecular detection for genetic changes in this cohort of patients by using the combination of MLPA and NGS was 44.6% (37 out of 83), while in 46 individuals (55.4%) we could not detect any alterations within the targeted genes.

### 3.3 Pathogenic/likely pathogenic/risk variants

Among 24 pathogenic/likely pathogenic/risk variants identified in this cohort, the most prevalent altered alleles were detected in *LRRK2*, *PRKN*, and *GBA* with frequencies of 38%, 29%, and 17%, respectively (Figure 1b). The details of pathogenic/likely pathogenic/risk variants are presented in Table 2.

**TABLE 2:** Pathogenic/likely pathogenic/risk factor mutations and variant of uncertain significance (VUS) identified in patients with early‐onset Parkinson's disease (EOPD)

Patient ID	Gender	Onset age	Family history	MDS‐UPDRS	Gene	Transmission	dbSNP	Transcript	Variant	ACMG Classification	ClinVar classification	Genetic diagnosis
PD043	F	41	N	6‐15‐21‐12	*PRKN*	Hom	–	NM_004562.3	Exon 5 deletion	PVS1, PS3, PS4	Not reported	Disease causing
PD099	M	33	N	2‐6‐12‐0	*PRKN*	Het	–	NM_004562.3	Exon 6 duplication	PVS1, PS3, PS4	Not reported	Disease causing
					*PRKN*	Het	–	NM_004562.3	Exon 2 deletion	PVS1, PS3, PS4	Not reported	
					*LRRK2*	Het	rs33949390	NM_198578.4	c.4883G>C (p.Arg1628Pro)	BS1, BP4, BP6, PP5	Conflicting interpretations of pathogenicity	
PD149	F	41	Y	9‐20‐33‐2	*PRKN*	Hom	–	NM_004562.3	Exon 4 deletion	PVS1, PS3, PS4	Not reported	Disease causing
					*MAPT*	Het	rs63750756	NM_016835.5	c.1788T>G (p.Asn596Lys)	PS1, PS3, PS4	Pathogenic	
					*LRRK2*	Hom	–	NM_198578.4	Exon 49 deletion	BP6	Not reported	
PD199	F	48	Y	11‐9‐13‐0	*VPS35*	Het	rs188286943	NM_018206.5	c.1858G>A (p.Asp620Asn)	PS1, PS3, PS4	Pathogenic	Disease causing
PD041	M	50	N	10‐16‐52‐11	*GBA*	Het	rs104886460	NM_000157.4	g.9069G>A (splice site mutation)	PVS1, PS3	Pathogenic/Likely pathogenic	Risk factor
PD068	M	30	N	16‐20‐67‐0	*PRKN*	Het	–	NM_004562.3	Exon 3 deletion	PVS1, PS3, PS4	Not reported	Carrier Risk factor
					*GBA*	Het	rs421016	NM_000157.4	c.1448T>C (p.Leu483Pro)	PP2, PP4	Pathogenic	
PD091	M	48	N	7‐15‐20‐5	*GBA*	Het	rs421016	NM_000157.4	c.1448T>C (p.Leu483Pro)	PP2, PP4	Pathogenic	Risk factor
PD158	M	40	N	3‐22‐31‐3	*GBA*	Het	rs80356772	NM_000157.4	c.1505G>A (p.Arg502His)	PS2, PP1, PP4	Pathogenic/Likely pathogenic	Risk factor
PD019	M	44	N	8‐6‐30‐2	*LRRK2*	Het	rs33949390	NM_198578.4	c.4883G>C (p.Arg1628Pro)	BS1, BP4, BP6, PP5	Conflicting interpretations of pathogenicity	Risk factor
PD023	F	41	Y	8‐4‐25‐3	*LRRK2*	Het	rs33949390	NM_198578.4	c.4883G>C (p.Arg1628Pro)	BS1, BP4, BP6, PP5	Conflicting interpretations of pathogenicity	Risk factor
PD061	F	36	N	0‐1‐17‐0	*LRRK2*	Het	rs33949390	NM_198578.4	c.4883G>C (p.Arg1628Pro)	BS1, BP4, BP6, PP5	Conflicting interpretations of pathogenicity	Risk factor
PD072	F	48	N	4‐8‐24‐0	*LRRK2*	Het	rs33949390	NM_198578.4	c.4883G>C (p.Arg1628Pro)	BS1, BP4, BP6, PP5	Conflicting interpretations of pathogenicity	Risk factor
PD080	M	38	N	1‐4‐25‐0	*LRRK2*	Het	rs33949390	NM_198578.4	c.4883G>C (p.Arg1628Pro)	BS1, BP4, BP6, PP5	Conflicting interpretations of pathogenicity	Risk factor
PD109	M	41	N	6‐7‐12‐0	*LRRK2*	Het	rs33949390	NM_198578.4	c.4883G>C (p.Arg1628Pro)	BS1, BP4, BP6, PP5	Conflicting interpretations of pathogenicity	Risk factor
PD123	M	49	N	5‐11‐26‐0	*LRRK2*	Het	rs33949390	NM_198578.4	c.4883G>C (p.Arg1628Pro)	BS1, BP4, BP6, PP5	Conflicting interpretations of pathogenicity	Risk factor
PD142	M	39	Y	8‐6‐10‐6	*LRRK2*	Het	rs33949390	NM_198578.4	c.4883G>C (p.Arg1628Pro)	BS1, BP4, BP6, PP5	Conflicting interpretations of pathogenicity	Risk factor
PD018	M	33	Y	7‐9‐43‐8	*PLA2G6*	Het	rs121908685	NM_003560.4	c.238G>A (p.Ala80Thr)	PM1, PM3	Pathogenic	Carrier
PD156	M	48	N	7‐20‐68‐6	*PINK1*	Het	–	NM_032409.3	Exon 1 deletion	PVS1, PS3, PS4	Not reported	Carrier
PD021	F	50	N	8‐4‐25‐3	*DNAJC13*	Het	–	NM_015268.4	c.5396T>C (p.lle1799Thr)	BP1	Not reported	–
PD041	M	50	N	10‐16‐52‐11	*ATP13A2*	Het	rs199661793	NM_022089.4	c.745G>A (p.Ala249Thr)	BP1	VUS	–
PD044	F	47	N	4‐11‐21‐4	*ATP13A2*	Het	rs202166353	NM_022089.4	c.3040G>A (p.Gly1014Ser)	BP1	VUS	–
PD049	M	48	N	11‐11‐9‐0	*DNAJC6*	Het	rs145175543	NM_014787.4	c.2044A>G (p.Ser682Gly)	BP1	VUS	–
PD056	M	46	N	3‐4‐18‐0	*MAPT*	Het	rs151115928	NM_016835.5	c.418C>T (p.Pro140Ser)	BP1	Benign	–
					*EIF4G1*	Het	rs200529085	NM_182917.4	c.3545C>G (p.Ala1182Gly)	BP1	Not reported	–
					*PINK1*	Het	rs35813094	NM_032409.3	c.1023G>A (p.Met341lle)	BP1	Not reported	–
PD063	F	40	N	7‐14‐16‐6	*DJ‐1*	Het	rs770946447	NM_007262.5	c.103G>A (p.Val35lle)	BP1	VUS	–
PD080	M	38	N	1‐4‐25‐0	*PINK1*	Het	–	NM_032409.3	c.1390C>T (p.Arg464Cys)	BP1	Not reported	–
PD089	F	47	N	6‐5‐11‐0	*PRKN*	Het	rs552077922	NM_004562.3	c.271G>A (p.Ala91Thr)	BP1	Not reported	–
PD091	M	48	N	7‐15‐20‐5	*ATP13A2*	Het	rs772243999	NM_022089.4	c.2557C>T (p.Arg853Cys)	BP1	VUS	–
PD092	M	26	N	2‐4‐15‐0	*ATP13A2*	Het	rs377186549	NM_022089.4	c.1202G>A (p.Cys401Tyr)	BP1	VUS	–
PD096	F	44	N	6‐16‐7‐7	*DNAJC6*	Het	rs145175543	NM_014787.4	c.2044A>G (p.Ser682Gly)	BP1	VUS	–
PD098	M	48	N	5‐6‐23‐3	*DNAJC6*	Het	rs145175543	NM_014787.4	c.2044A>G (p.Ser682Gly)	BP1	VUS	–
PD103	M	46	N	6‐6‐15‐2	*GCH1*	Het	rs756782285	NM_000161.3	c.170G>A (p.Arg57Gln)	BP1	Not reported	–
PD110	M	50	N	5‐8‐27‐0	*VPS13C*	Het	rs568860952	NM_020821.3	c.7559C>G (p.Ala2520Gly)	BP1	Not reported	–
PD140	F	47	N	7‐14‐59‐3	*EIF4G1*	Het	rs200529085	NM_182917.4	c.3545C>G (p.Ala1182Gly)	BP1	Not reported	–
PD148	M	40	N	15‐14‐24‐10	*FBXO7*	Het	rs548204763	NM_012179.4	c.587A>G (p.Asn196Ser)	BP1	VUS	–
PD150	F	37	N	7‐23‐46‐8	*LRRK2*	Het	rs281865042	NM_198578.4	c.1847A>G (p.Lys616Arg)	BP1	Not reported	–
PD158	M	40	N	3‐22‐31‐3	*EIF4G1*	Het	rs143014570	NM_182917.4	c.1331C>T (p.Thr444Met)	BP1	Not reported	–
PD168	M	42	N	22‐25‐58‐9	*DNAJC13*	Het	rs553930800	NM_015268.4	c.5646G>C (p.Met1882lle)	BP1	Not reported	–
PD183	M	50	N	14‐19‐32‐8	*VPS13C*	Het	rs11629598	NM_020821.3	c.4354A>G (p.lle1452Val)	BP1	Not reported	–
PD192	M	48	N	12‐23‐40‐7	*EIF4G1*	Het	rs112809828	NM_182917.4	c.3988A>G (p.Met1330Val)	BP1	Not reported	–
PD193	M	33	N	2‐9‐47‐3	*EIF4G1*	Het	rs746958243	NM_182917.4	c.1223C>G (p.Pro408Arg)	BP1	Not reported	–
PD210	M	37	N	4‐9‐21‐0	*LRRK2*	Het	rs202157354	NM_198578.4	c.158A>G (P.Lys53Arg)	BP1	VUS	–

Abbreviations: ACMG, American College of Medical Genetics; F, female; Hom/Het, homogenous/heterogenous; M, male; Family history, Y (yes)/N (no); MDS‐UPDRS, MDS‐Unified Parkinson's Disease Rating Scale (part 1‐part 2‐part 3‐part4).

Alterations of *LRRK2* were the most prevalent in the 83 patients with EOPD. Nine patients had *LRRK2* c.4883G>C (p.Arg1628Pro), one with the c.1847A>G (p.Lys616Arg) (PD150), one with c.158A>G (p.Lys53Arg) (PD210), and one with homozygous deletion of exon 49 (PD149). When compared with the unidentified group, patients with PD carrying *LRRK2* c.4883G>C (p.Arg1628Pro) had a younger age of onset and significantly lower MDS‐UPDRS scores in all four parts recorded (Table 3).

**TABLE 3:** Comparison of clinical characteristics between LRRK2 c.4883G>C and unidentified patients with Parkinson's disease (PD)

Characteristics	*LRRK2* Arg1628Pro N=9	Unidentified N=46	*p*‐Value
Age of onset (mean±SD)	41.00±5.29	43.41±6.18	.24
Gender, N (%)			
Male	6 (66.67)	22 (47.82)	.30
Female	3 (33.33)	24 (52.17)	
Family history of PD, N (%)			
Yes	2 (22.22)	7 (15.22)	.60
No	7 (77.78)	39 (84.78)	
MDS‐UPDRS part I (mean±SD)	4.67±3.12	7.98±4.77	.02^a^
MDS‐UPDRS part II (mean±SD)	5.89±2.80	10.67±5.75	.001^a^
MDS‐UPDRS part III (mean±SD)	20.11±7.41	31.39±13.20	.001^a^
MDS‐UPDRS part IV (mean±SD)	1.22±2.11	2.82±3.29	.03^a^

Abbreviation: MDS‐UPDRS, MDS‐Unified Parkinson's Disease Rating Scale.

^a^ Statistically significant.

Mutations of *PRKN* in four patients were all large genetic arrangements, including deletions of exon 2, 3, 4, 5, and duplication of exon 6. Two patients had homozygous *PRKN* deletion (PD43: exon 5, PD149: exon 4). Patient PD 99 was confirmed to have compound heterozygous rearrangements in *PRKN* by genetic analysis in his family (data not shown).

Our study identified four male *GBA1*‐related patients with EOPD, including heterozygous splice‐site (g.9069G>A), c.1448T>C (p.Leu483Pro), and c.1505G>A (p.Arg502His). These patients had no family history of PD, and we found that the g.9069G>A carrier (PD41) rapidly progressed to Hoehn‐Yahr stage 3 with motor complications after 6 years of disease.

Furthermore, two heterozygous missense mutations were found in *PLA2G6* (c.238G>A, p.Ala80Thr) and *VPS35* (c.1858G>A, p.Asp620Asn), which have been reported as pathogenic. The heterozygous *PINK1* deletion of exon 1 (PD156) was found in a recently diagnosed 48‐year‐old male with depressed mood; MDS‐UPDRS score 7‐20‐68‐6 each part, respectively, and cognitive impairment (MoCA of 23).

Patient PD68 carrying *PRKN* deletion of exon 3 and *GBA1* p.Leu483Pro had earliest disease onset at the age of 30, and highly pronounced disturbances in mood, motor symptoms (MDS‐UPDRS each part were 16‐20‐67‐12, respectively).

### 3.4 Variant of uncertain significance

Twenty‐five variant of uncertain significance (VUS) were identified according to ACMG criteria in 23 patients with PD (27.7% of the participants). The details of these variants are listed in Table 2. All the variants were missense heterozygous. The distribution of variants by genes is shown in Figure 1c. These variants were identified mainly in the *EIF4G1*, *ATP13A2*, *DNAJC13*, and *DNAJC6* genes. No genetic alterations were identified in *SNCA, UCHL1, SYNJ11, HTRA2*, and *CHCHD2*.

## 4 DISCUSSION

The development of NGS in Vietnam has allowed comprehensive genetic studies of multiple pathogenic conditions (M. D. Do et al., January, 2022; H. T. Nguyen et al., 2020; H.‐N. Nguyen et al., 2021; Nguyen‐Le, 2022). Analyzing the spectrum of PD‐related genes in different ethnicities is becoming important to the understanding of the genetic mechanism underlying the disease. In this study, we determined the mutational spectrum of 20 known PD‐associated genes in a cohort of Kinh Vietnamese patients diagnosed with EOPD, and identified 37 out of 83 (45%) patients carrying variants in *LRRK2, PRKN, EIF4G1, ATP13A2, GBA1, DNAJC6, PINK1, DNAJC13, MAPT, VPS13C, DJ‐1, FBXO7, GCH1, PLA2G6*, and *VPS35*.

The *LRRK2* gene (leucine‐rich repeat kinase 2) encodes Lrrk2 containing ARM (armadillo repeat motifs), ANK (ankyrin repeat), LRR (leucine‐rich repeat), ROC (Ras of complex proteins; GTPase), COR (C‐terminal of ROC), MAP‐KKK (mitogen‐activated kinase kinase kinase), and WD40 domains (Gasser, 2011). It is the best‐known cause of autosomal dominant PD, accounting for 5% of familial and 1% of sporadic cases (Kestenbaum & Alcalay, 2017). The p.Gly2019Ser mutation located in the MAP‐KKK kinase domain is common in Caucasians, accounting for 1% of sporadic cases (Bardien et al., 2011; Haugarvoll & Wszolek, 2009), while p.Gly2385Arg and p.Arg1628Pro mutations are risk variants found in 3%–4% of healthy individuals and 6%−8% of patients with PD in some Asian populations (Ross et al., 2008). The *LRRK2* p.Arg1628Pro variant is mostly identified as a secondary susceptibility genetic factor, especially in patients of Chinese descent, conferring a twofold risk of developing PD, with typical late‐onset L‐dopa‐responsive clinical phenotype in carriers (Cao et al., 2007; Liang et al., 2018; Ross et al., 2008; Zhao et al., 2020). Penetrance of *LRRK2* is age‐dependent and widely variably, with estimated rate ranging from 30% to 74% (Ozelius et al., 2006; Schneider & Alcalay, 2020). Our present study found that the proportion of patients carrying the *LRRK2* variants was 15% (12 out of 83), higher compared to either Korean (8.6%; six out of 70) or Chinese population (9.2%; 22 out of 240) (Li et al., 2020; Youn et al., 2019). Interestingly, we found that *LRRK2* p.Arg1628Pro was the most frequent variant in Vietnamese patients with EOPD, whereas this variant was described mostly in patients with late‐onset PD (Li et al., 2020; S.‐Y. Lim et al., 2019; Zhang et al., 2017). Arginine in codon 1628 is in the COR domain of the Lrrk2 protein and highly conserved across species, emphasizing the importance of this residue to protein function. It is postulated that the substitution of a neutral nonpolar proline at this position may cause a conformational alteration misleading to Lrrk2 dimerization (Ross et al., 2008). Further studies to elucidate how *LRRK2* p.Arg1628Pro could trigger the onset of PD are required to fully understand whether it was a risk variant or a pathogenic mutation with low penetrance in Asian. In this study, we described that *LRRK2* variants carriers had identical clinical features of idiopathic PD similar to previous reports (Alcalay et al., 2009; Gan‐Or et al., 2015; Liang et al., 2018; Pulkes et al., 2014). Lysine 616 is one among the conserved amino acid of Lrrk2. The missense *LRRK2* p.Lys616Arg mutation was first identified in a Chinese family as dominant in a late‐onset form of PD, with slow progression and no reported motor complications (Wang et al., 2010). The patient carrying this variant in our study (PD150) exhibited distinct clinical manifestations. Further studies on *LRRK2* variants are needed to explain its role in the pathophysiology of PD.

Parkin plays critical role as ubiquitin ligase E3, protecting against toxicity and oxidative stress (Castelo Rueda et al., 2021). Mutated *PRKN* was previously reported to be the most common genetic cause of early onset typical PD (Kitada et al., 1998). More than 130 variants have been described, mostly related to copy number variants either large deletions or duplications of entire exons. The mutation frequency of *PRKN* occurs various on different populations (Kilarski et al., 2012; Li et al., 2020; Lin et al., 2019). We reported herein six *PRKN* variant carriers (7%), including deletion, duplication, and point mutation. Exon deletion expanding from exon 2 to exon 5 was the most observed type, similar to previous studies (Guo et al., 2015; Jiang et al., 2020). No family history of disease was detected in most of these cases. Intriguingly, we showed that those three out of five patients carrying *PRKN* variants had cognitive impairment (MoCA score less than 26), which was unusual as other findings.

The microtubule‐associated protein tau (MAPT) plays an important role in tubulin polymerization, stabilization of microtubules, and maintaining cellular processes. *MAPT* p.Asn596Lys has been reported in patients diagnosed with pallido‐ponto‐nigral degeneration (Clark et al., 1998; Yasuda et al., 1999) and has been confirmed as a pathogenic mutation. The patient carrying *PRKN* deletion of exon 4, *LRRK2* deletion of exon 49, and *MAPT* p.Asn596Lys had dominant motor disturbances (high MDS‐UPDRS score of part III), but without the presence of apathy as previously reported (Espay & Litvan, 2011; Yang et al., 2015).

Variants in the glucocerebrosidase gene (*GBA1*) are common and important genetic susceptibility factors for PD (J. Do et al., 2019). We identified four heterozygous carriers with the frequency of 5%, as relevant to J. L. Lim et al. (2022), including one carried g.9069G>A (c.115+1G>A), one carried p.Arg502His, and two carried p.Leu483Pro. Notably, these rare variants had been identified as being pathogenic in Gaucher disease and as genetic risk factors for PD in the heterozygous state (Malek et al., 2018). *GBA1*‐related patients with PD have earlier age at onset, higher prevalence of the postural instability, gait‐difficulty phenotype, worse motor symptoms, more frequent non‐motor symptoms, rapid progression, and reduced survival compared with non‐*GBA1*‐mutated patients with PD (Brockmann et al., 2015; Malek et al., 2018; Maple‐Grødem et al., 2021; Stoker et al., 2020). *GBA1* p.Leu483Pro is among the three most common variants in patients with PD (Guadagnolo et al., 2021; Huang et al., 2011; J. L. Lim et al., 2022; Petrucci et al., 2020; Ren et al., 2022; Wu et al., 2007), whereas the splice‐site variant g.9069G>A (c.115+1G>A) has previously been identified in both PD subjects and asymptomatic carriers (Aslam et al., 2021; Sato et al., 2005). Compared with patients who did not carry a *GBA1* mutation, those with *GBA1* mutations were male and presented earlier onset and cognitive changes (MoCA: 24.25±3.77) (Sidransky et al., 2009) but no family history was detected. From our observation, the presence of *GBA1* variants (especially *GBA1* p.Leu483Pro) may accelerate the disease progression (Cilia et al., 2016; Liu et al., 2016). Previous experimental data have shown that GCase and α‐synuclein form a bidirectional pathogenic loop (Mazzulli et al., 2011) in which the functional loss of GCase caused by the *GBA1* variant integrates the degradation of lysosomal α‐syn, leading to the accumulation of α‐syn; α‐syn aggregation inhibits the lysosomal activity of GCase. However, the association between the severity of the *GBA1* variant and GCase activity level has not been elucidated (Petrucci et al., 2020). Therefore, the effect of *GBA1* variants on PD pathogenesis is crucial for detailed investigation.

*PINK1* mutations are the second most common cause of EOPD and autosomal recessive PD. The frequency of *PINK1* genetic alterations in our study was 3.6% (three out of 83). The heterozygous *PINK1* deletion of exon 1 carrier (PD156) had appropriate features as previously reported (Guadagnolo et al., 2021), especially the depression mood and cognitive impairment (MoCA score: 23 points).

The two known mutants on *PLA2G6* p.Ala80Thr and *VPS35* p.Asp620Asn were identified in our cohort with the frequency of 1.2% (one out of 83 for each) presented similar characteristics as previously reported (Agarwal et al., 2012, Magrinelli et al., 2022; Yoshino et al., 2022), except the early age at onset.

In conclusion, seven patients (8.4%) carried pathogenic or likely pathogenic variants in known PD genes in our patients with EOPD. Additionally, 13.3% of patients (11/83) carried risk variants in either *LRRK2* or *GBA1*, and 19 patients (22.9%) had rare variants of uncertain significance. Our findings contribute a primary understanding of the genetic spectrum of Vietnamese EOPD, indicating that specific pathogenic/likely pathogenic variants may underlie different phenotypic manifestations, and the pathogenicity of numerous either rare variants or high‐risk variants should be further considered. However, our data have some limitations: (i) the sample size was relatively small; (ii) the number of subjects carrying variants of different severity may conceal additional significant differences; and (iii) we were unable to obtain data on the longitudinal progression of motor and key non‐motor symptoms, which will be necessary for future research.

## AUTHOR CONTRIBUTIONS

Thao Phuong Mai and Minh Duc Do designed the study. Tai Ngoc Tran and Tuan Van Le recruited the patients. An Bac Luong, Linh Hoang Gia Le, Niem Thanh Van Vo, Khuong Thai Le, and Hoang Anh Vu performed the genetic sequencing. Minh Duc Do, Thao Phuong Mai, Tai Ngoc Tran, Thuc‐Nhi Nguyen Le, and Hoang Anh Vu analyzed the data. Thao Phuong Mai and Minh Duc Do wrote the manuscript.

## CONFLICT OF INTEREST STATEMENT

The authors declare no conflict of interest.

### PEER REVIEW

The peer review history for this article is available at https://publons.com/publon/10.1002/brb3.2950.

## Data Availability

Raw data supporting the conclusion of this manuscript are available upon request, contact drmaithao@ump.edu.vn.
